# O12 A rare mimic of inflammatory arthritis

**DOI:** 10.1093/rap/rkab067.011

**Published:** 2021-10-19

**Authors:** Hafiz Muhammad Umair, Muhammad Farooq Kazmi

**Affiliations:** Sheffield Teaching Hospitals, Sheffield, United Kingdom

## Abstract

**Case report - Introduction:**

Melorheostosis is a very rare benign bone disorder involving sclerosing hyperostosis. The name derives from the Greek terms Melos ‘limb’, rheos ‘flow’ and osteon ‘bone’. The incidence of melorheostosis is 0.9 cases per million population, and in the majority of cases is diagnosed before the age of 20 years. It presents with pain, deformities, limitations of a range of motion, contractures, muscle atrophy, and limb swelling. We present a case of a 33-year-old lady who was referred with a history of pain and swelling of the right knee.

**Case report - Case description:**

This 33-year-old lady of Nigerian origin was referred to the rheumatology department with a 7-year history of fluctuating pain and swelling in the right knee with the possibility of inflammatory arthritis. Prior to this, she had been seen by the local orthopaedic team with subsequent input sought from the regional specialist orthopaedic bone unit in Birmingham. The synovial biopsies did not show any evidence of inflammation or pigmented villo-nodular synovitis (PVNS). Prior to moving to the UK, she had been assessed in Germany and Dubai due to knee pain and swelling with a diagnosis of the unclear bone-related condition and fibromyalgia. Clinically the right knee had a chronic cool swelling with reduced range of movement but no synovitis and no signs of inflammatory arthritis in other joints.  There were no features of seronegative inflammatory arthritis or connective tissue diseases.  Investigations showed weak positive ANA with normal CRP, bone profile, FBC, urea and electrolytes, with negative rheumatoid factor and anti-CCP antibody. X-ray knee arranged, showed a flowing periosteal thickening medial cortices of the femur and tibia consistent with ‘melorheostosis’. MRI right knee also confirmed the same diagnosis. Imaging of the upper limbs, spine and pelvis showed no involvement. Input from the metabolic bone health team was arranged who did not deem bisphosphonates useful in her case. Physiotherapy input was arranged to improve her leg posture and movements along with appropriate patient education and analgesia titration.

**Case report - Discussion:**

We present a rare mimic of inflammatory arthritis. Initially referred by GP to orthopaedic team who considered PVNS which was ruled out by biopsies, then next question was about inflammatory arthritis due to recurrent history of knee pain and swelling which is valid differential for monoarthritis. Rheumatology assessment noted a puffy knee but no effusion to aspirate, x-ray imaging was diagnostic for melorheostosis due to the typical presence of ‘dripping wax appearance’.

As rheumatologists while assessing joints, genetic and metabolic causes need to be considered besides inflammatory and infective causes. A thorough history remains essential along with interaction with radiology colleagues which is the main learning point.

**Case report - Key learning points:**

1. Non-inflammatory conditions like melorheostosis can mimic inflammatory arthritis
2. Simple imaging like X-rays and discussion with radiology colleagues are often underrated.

